# Supplementary material for: The association between diet and mental health and wellbeing in young adults within a biopsychosocial framework
Source: PLoS One. 2021 Jun 3;16(6):e0252358. doi: 10.1371/journal.pone.0252358 (PMC8174719; doi:10.1371/journal.pone.0252358)
Supplement: S1 File — (PDF) [file pone.0252358.s001.pdf]

Food and Mood Survey

Start of Block: Consent Form (not included here)

Thank you for deciding to participate in this study, we greatly appreciate it!

In this first part of the survey, you will be asked about your mental wellbeing. Please note that some of the questions ask about a certain time frame (e.g. ***in the last 2 weeks*** or ***during the past month***), others ask if you have ***ever*** experienced certain things. Please make sure you read the questions carefully.

Remember that answering these questions is voluntary. If you choose not to answer a certain question, please check the box “prefer not to say” in order to move on to the next question.

Some of the questions might make you sad or make you remember difficult times in your life. Should you experience any stress as a result from this survey, please do not hesitate to contact either one of the study staff or UBC Counselling Services located in Room 1040 in Brock Hall, 1874 East Mall or Empower Me1-844-741-6389 (toll-free). For crisis or after-hours support services, please contact the service you are most comfortable with from this list.

In general, would you say your quality of life is:

- ○ Excellent
- ○ Very good
- ○ Good
- ○ Fair
- ○ Poor
- ○ Prefer not to say

In general, how would you rate your satisfaction with your social activities and relationships?

- ○ Excellent
- ○ Very good
- ○ Good
- ○ Fair
- ○ Poor
- ○ Prefer not to say

I am able to balance my academic time (in class, study time, etc.) and non-academic time (work, exercise, socializing, care for dependents etc.)

- Strongly agree
- Agree
- Somewhat agree
- Somewhat disagree
- Disagree
- Strongly disagree
- Prefer not to say

I am confident that I will succeed in all of my courses

- Strongly agree
- Agree
- Somewhat agree
- Somewhat disagree
- Disagree
- Strongly disagree
- Prefer not to say

Within the ***last 12 months***, how would you rate the overall level of stress you have experienced?

- ○ No stress
- ○ Less than average stress
- ○ Average stress
- ○ More than average stress
- ○ Tremendous stress
- ○ Prefer not to say

Over the ***last 2 weeks***, how often have you been bothered by any of the following problems?

	Not at all	Several days	More than half the days	Nearly every day	Prefer not to say
Little interest or pleasure in doing things	○	○	○	○	○
Feeling down, depressed, or hopeless	○	○	○	○	○
Trouble falling or staying asleep, or sleeping too much	○	○	○	○	○
Feeling tired or having little energy	○	○	○	○	○
Poor appetite or overeating	○	○	○	○	○
Feeling bad about yourself or that you are a failure or have let yourself or your family down	○	○	○	○	○

Trouble concentrating on things, such as reading the newspaper or watching television	○	○	○	○	○
Moving or speaking so slowly that other people could have noticed. Or the opposite – being so fidgety or restless that you have been moving around a lot more than usual	○	○	○	○	○
Thoughts that you would be better off dead or of hurting yourself	○	○	○	○	○

Thinking about the problems you checked off in the previous question, how difficult have these problems made it for you to do your work, take care of things at home, or get along with other people?

- ○ Not difficult at all
- ○ Somewhat difficult
- ○ Very difficult
- ○ Extremely difficult
- ○ Prefer not to say

Over the ***last 2 weeks***, how often have you been bothered by the following problems?

	Not at all	Several days	More than half the days	Nearly every day	Prefer not to say
Feeling nervous, anxious, or on edge	○	○	○	○	○
Not being able to stop or control worrying	○	○	○	○	○
Worrying too much about different things	○	○	○	○	○
Trouble relaxing	○	○	○	○	○
Being so restless that it's hard to sit still	○	○	○	○	○
Becoming easily annoyed or irritable	○	○	○	○	○
Feeling afraid as if something awful might happen	○	○	○	○	○

Have you ***ever*** been diagnosed or treated ***by a professional*** for any of the following? Check all that apply.

- ☐ Depression
- ☐ Anxiety
- ☐ Eating disorder
- ☐ Panic attacks
- ☐ Substance use
- ☐ Phobia
- ☐ Obsessive compulsive disorder
- ☐ Schizophrenia/Psychosis
- ☐ Personality disorder
- ☐ Other, please specify: ____________________
- ☐ I have never been diagnosed with any of the above mentioned disorders
- ☐ Prefer not to say

How old were you when you were ***first*** diagnosed with {automatic entry from previous questions}?

____

Have you ***ever*** struggled with any of the following mental health issues that were ***not*** diagnosed by a professional? Check all that apply.

- ☐ Depression
- ☐ Anxiety
- ☐ Eating disorder
- ☐ Panic attacks
- ☐ Substance use
- ☐ Phobia
- ☐ Obsessive compulsive disorder
- ☐ Schizophrenia/Psychosis
- ☐ Personality disorder
- ☐ Other, please specify: ____________________
- ☐ I have never struggled with any of the above mentioned issues
- ☐ Prefer not to say

How old were you when you ***first*** struggled with {automatic entry from previous question}?

____

Does anyone in your family have a history of mental illness? Check all that apply.

- [ ] Mother
- [ ] Father
- [ ] Brother(s)
- [ ] Sister(s)
- [ ] Grandmother(s)
- [ ] Grandfather(s)
- [ ] Aunt(s)
- [ ] Uncle(s)
- [ ] Cousin(s)
- [ ] Others, please specify: ____________________
- [ ] Don't know
- [ ] No one in my family has a history of mental illness
- [ ] Prefer not to say

Are you satisfied with your eating patterns?

- ◯ Yes
- ◯ No
- ◯ Prefer not to say

Do you ever eat in secret?

- ◯ Yes
- ◯ No
- ◯ Prefer not to say

Does your weight affect the way you feel about yourself?

- ◯ Yes
- ◯ No
- ◯ Prefer not to say

Have any family members of your family suffered with an eating disorder?

- ◯ Yes
- ◯ No
- ◯ Don't know
- ◯ Prefer not to say

How do you describe your weight?

- Very underweight
- Slightly underweight
- About the right weight
- Slightly overweight
- Very overweight
- Prefer not to say

How satisfied are you with your current weight?

- Not satisfied at all
- Slightly unsatisfied
- Somewhat satisfied
- Very satisfied
- Extremely satisfied
- Prefer not to say

Are you trying to do any of the following about your weight?

- ○ I am not trying to do anything about my weight
- ○ Stay the same weight
- ○ Lose weight
- ○ Gain weight
- ○ Prefer not to say

Thinking back over the ***past 30 days***, how many cigarettes (the kind that come in a pack or roll-your-own) did you smoke in a normal week?

- ○ None
- ○ 1-5/week
- ○ 6-15/week
- ○ about a pack/week
- ○ more than a pack/week
- ○ Prefer not to say

Thinking back over the ***past 30 days***, how often did you consume marijuana in a normal week?

- None
- Less than 1 day a week
- 1-2 days/week
- 3-6 days/week but not every day
- Every day
- Prefer not to say

Thinking back over the ***past 30 days***, how often did you consume alcohol in a normal week?

- Never
- Less than 1 day a week
- 1-2 days/week
- 3-6 days/week but not every day
- Every day
- Prefer not to say

In the ***past year***, have any of these events occurred in your life? Check all that apply.

- ☐ Death of a close family member
- ☐ Death of a close friend
- ☐ Divorce between parents
- ☐ Serious legal problems
- ☐ Major personal injury or illness
- ☐ Responsibilities for others such as children/spouse
- ☐ Threat to major source of income
- ☐ Difficulty with roommate(s)
- ☐ Change in health of a family member
- ☐ Pregnancy
- ☐ Sexual problems
- ☐ Serious disagreements with parents
- ☐ Change in lifestyle for financial reasons
- ☐ Difficulty in identifying a major
- ☐ Serious argument with close family member
- ☐ Problems with a girlfriend or boyfriend
- ☐ Having to repeat a course
- ☐ Increased workload at school

- [ ] Outstanding personal achievement
- [ ] First semester in college
- [ ] Change in living conditions
- [ ] Serious disagreements with an instructor
- [ ] Lower grades than expected
- [ ] Change in sleeping habits
- [ ] Change in social habits
- [ ] Change in eating habits
- [ ] Chronic car problems
- [ ] Change in number of family get togethers
- [ ] Too many missed classes
- [ ] Change in plans for a major
- [ ] Dropped more than one class
- [ ] Minor traffic violations
- [ ] Prefer not to say

In general, how would you rate your physical health?

- ◯ Excellent
- ◯ Very good
- ◯ Good
- ◯ Fair
- ◯ Poor
- ◯ Prefer not to say

On how many of the ***past 7 days*** did you participate in vigorous exercise for at least 20 minutes or moderate exercise for at least 30 minutes? Vigorous exercise are activities that take hard physical effort and make you breathe much harder than normal. Moderate exercise are activities that take moderate physical effort and make you breathe somewhat harder than normal.

- ◯ Never
- ◯ 1 day/week
- ◯ 2 days/week
- ◯ 3 days/week
- ◯ 4 days/week
- ◯ 5 days/week
- ◯ 6 days/week
- ◯ Every day
- ◯ More than once a day (i.e. more than 7 times/week)
- ◯ Prefer not to say

On how many of the ***past 7 days*** did you get enough sleep so that you felt rested when you woke up in the morning?

- ◯ Never
- ◯ 1 day/week
- ◯ 2 days/week
- ◯ 3 days/week
- ◯ 4 days/week
- ◯ 5 days/week
- ◯ 6 days/week
- ◯ Every day
- ◯ Prefer not to say

Do you currently take any medication for mental health issues?

- ◯ Yes
- ◯ No
- ◯ Prefer not to say

Please tell us more about the medication you take for mental health issues:

- ◯ Name of the medication ____
- ◯ Dosage you take (e.g. one capsule of 20mg/day) ____

Great, part one is done! Now we will move on to the second part of the survey.

The following questions will ask about your diet, food allergies, and more. Please answer every question, failure to do so will lead your responses to be unusable for this study. If you are unsure about the answer, try to give your best estimate.

The questions are about foods you ate and drinks you drank ***during the past month***, that is, the past 30 days. When answering, please include meals and snacks at home, at work or school, in restaurants and anyplace else.

During the ***past month***, how often did you eat hot or cold cereals? Check one.

- ○ Never
- ○ 1x/month
- ○ 2-3x/month
- ○ 1x/week
- ○ 2x/week
- ○ 3-4x/week
- ○ 5-6x/week
- ○ 1x/day
- ○ 2 or more times/day

During the ***past month***, what kind of cereal did you usually eat?

____

If there was another kind of cereal that you usually ate during the ***past month***, what kind was it?

____

During the ***past month***, how often did you have any dairy milk (either to drink or on cereal)? Include regular milks, chocolate or flavored milks, lactose-free milk, buttermilk. Please do not include small amounts of milk in coffee or tea. Do not include soy milk or other non-dairy milks such as almond milk. Check one.

- ○ Never
- ○ 1x/month
- ○ 2-3x/month
- ○ 1x/week
- ○ 2x/week
- ○ 3-4x/week
- ○ 5-6x/week
- ○ 1x/day
- ○ 2-3x/day
- ○ 4-5x/day
- ○ 6 or more times/day

During the ***past month***, what kind of dairy milk did you usually drink? Check one.

- ○ Whole or regular milk
- ○ 2% fat or reduced-fat milk
- ○ 1% or 0.5% or low-fat milk
- ○ Fat-free, skim or non-fat milk
- ○ Other kind of milk, please specify: ______________________

During the ***past month***, how often did you have any non-dairy milk such as soy milk, almond milk, rice milk etc. (either to drink or on cereal)? Please do not include small amounts of milk in coffee or tea. Check one.

- ○ Never
- ○ 1x/month
- ○ 2-3x/month
- ○ 1x/week
- ○ 2x/week
- ○ 3-4x/week
- ○ 5-6x/week
- ○ 1x/day
- ○ 2 or more times/day

During the ***past month***, how often did you drink regular soda or pop that contains sugar? Do not include diet soda. Check one.

- ◯ Never
- ◯ 1x/month
- ◯ 2-3x/month
- ◯ 1x/week
- ◯ 2x/week
- ◯ 3-4x/week
- ◯ 5-6x/week
- ◯ 1x/day
- ◯ 2-3x/day
- ◯ 4-5x/day
- ◯ 6 or more times/day

During the ***past month***, how often did you drink 100% pure fruit juices such as orange, mango, apple, grape and pineapple juice? Do <u>not</u> include fruit-flavored drinks with added sugar or fruit juice you made at home and added sugar to. Check one.

- ○ Never
- ○ 1x/month
- ○ 2-3x/month
- ○ 1x/week
- ○ 2x/week
- ○ 3-4x/week
- ○ 5-6x/week
- ○ 1x/day
- ○ 2-3x/day
- ○ 4-5x/day
- ○ 6 or more times/day

During the ***past month***, how often did you drink coffee or tea that had sugar or honey added to it? Include coffee and tea you sweetened yourself and pre-sweetened tea and coffee drinks

such as Arizona Iced Tea or Frappuccino. Do <u>not</u> include artificially sweetened coffee or diet tea. Check one.

- ○ Never
- ○ 1x/month
- ○ 2-3x/month
- ○ 1x/week
- ○ 2x/week
- ○ 3-4x/week
- ○ 5-6x/week
- ○ 1x/day
- ○ 2-3x/day
- ○ 4-5x/day
- ○ 6 or more time

During the ***past month***, how often did you drink sweetened fruit drinks, sports or energy drinks, such as Kool-Aid, lemonade, Hi-C, cranberry drink, Gatorade, Red Bull or Vitamin Water?

Include fruit juices you made at home and added sugar to. Do <u>not</u> include diet drinks or artificially sweetened drinks. Check one.

- ○ Never
- ○ 1x/month
- ○ 2-3x/month
- ○ 1x/week
- ○ 2x/week
- ○ 3-4x/week
- ○ 5-6x/week
- ○ 1x/day
- ○ 2-3x/day
- ○ 4-5x/day
- ○ 6 or more times/day

During the ***past month***, how often did you eat fruit? Include fresh, frozen or canned fruit. Do <u>not</u> include juices. Check one.

- ◯ Never
- ◯ 1x/month
- ◯ 2-3x/month
- ◯ 1x/week
- ◯ 2x/week
- ◯ 3-4x/week
- ◯ 5-6x/week
- ◯ 1x/day
- ◯ 2 or more times/day

During the ***past month***, how often did you eat a green leafy or lettuce salad, with or without other vegetables? Check one.

- ◯ Never
- ◯ 1x/month
- ◯ 2-3x/month
- ◯ 1x/week
- ◯ 2x/week
- ◯ 3-4x/week
- ◯ 5-6x/week
- ◯ 1x/day
- ◯ 2 or more times/day

During the ***past month***, how often did you eat any kind of fried potatoes, including French fries, home fries, or hash brown potatoes? Check one.

- ○ Never
- ○ 1x/month
- ○ 2-3x/month
- ○ 1x/week
- ○ 2x/week
- ○ 3-4x/week
- ○ 5-6x/week
- ○ 1x/day
- ○ 2 or more times/day

During the ***past month***, how often did you eat any other kind of potatoes, such as baked, boiled, mashed potatoes, sweet potatoes, or potato salad? Check one.

- ○ Never
- ○ 1x/month
- ○ 2-3x/month
- ○ 1x/week
- ○ 2x/week
- ○ 3-4x/week
- ○ 5-6x/week
- ○ 1x/day
- ○ 2 or more times/day

During the ***past month***, how often did you eat cooked dried beans or canned beans and legumes such as baked beans, pinto beans, kidney beans, lima beans, lentils, soybeans, chickpeas, or refried beans? Do <u>not</u> include green beans. Check one.

- ○ Never
- ○ 1x/month
- ○ 2-3x/month
- ○ 1x/week
- ○ 2x/week
- ○ 3-4x/week
- ○ 5-6x/week
- ○ 1x/day
- ○ 2 or more times/day

During the ***past month***, how often did you eat brown rice or other cooked whole grains, such as quinoa, barley, bulgur, cracked wheat, or millet? Do <u>not</u> include white rice. Check one.

- Never
- 1x/month
- 2-3x/month
- 1x/week
- 2x/week
- 3-4x/week
- 5-6x/week
- 1x/day
- 2 or more times/day

During the ***past month***, not including what you just told us about (green salads, potatoes, cooked dried beans etc.) how often did you eat other vegetables? Check one.

- Never
- 1x/month
- 2-3x/month
- 1x/week
- 2x/week
- 3-4x/week
- 5-6x/week
- 1x/day
- 2 or more times/day

During the ***past month***, how often did you eat nuts and seeds such as peanuts, cashews, walnuts, pecans, sunflower seeds or pumpkin seeds? Check one.

- ○ Never
- ○ 1x/month
- ○ 2-3x/month
- ○ 1x/week
- ○ 2x/week
- ○ 3-4x/week
- ○ 5-6x/week
- ○ 1x/day
- ○ 2 or more times/day

During the ***past month***, how often did you eat pizza? Include frozen pizza, fast food pizza, and homemade pizza. Check one.

- ○ Never
- ○ 1x/month
- ○ 2-3x/month
- ○ 1x/week
- ○ 2x/week
- ○ 3-4x/week
- ○ 5-6x/week
- ○ 1x/day
- ○ 2 or more times/day

During the ***past month***, how often did you eat tomato sauces such as with spaghetti or noodles or mixed into foods such as lasagna? Do <u>not</u> include tomato sauce on pizza. Check one.

- ○ Never
- ○ 1x/month
- ○ 2-3x/month
- ○ 1x/week
- ○ 2x/week
- ○ 3-4x/week
- ○ 5-6x/week
- ○ 1x/day
- ○ 2 or more times/day

During the ***past month***, how often did you eat any kind of cheese? Include cheese as a snack, cheese on burgers, sandwiches, and cheese in foods such as lasagna quesadillas or casseroles. Do not include cheese on pizza. Check one.

- ◯ Never
- ◯ 1x/month
- ◯ 2-3x/month
- ◯ 1x/week
- ◯ 2x/week
- ◯ 3-4x/week
- ◯ 5-6x/week
- ◯ 1x/day
- ◯ 2 or more times/day

During the ***past month***, how often did you eat yogurt, Greek yogurt or other dairy products? Do not include fluid dairy milk or cheese. Check one.

- ◯ Never
- ◯ 1x/month
- ◯ 2-3x/month
- ◯ 1x/week
- ◯ 2x/week
- ◯ 3-4x/week
- ◯ 5-6x/week
- ◯ 1x/day
- ◯ 2 or more times/day

During the ***past month***, how often did you eat red meat, such as beef, pork ham, or sausage? Include red meat you had in sandwiches, lasagna, stew, and other mixtures. Red meats may

also include veal, lamb, and any lunch meats made with these meats. Do <u>not</u> include chicken, turkey, fish or seafood. Check one.

- ○ Never
- ○ 1x/month
- ○ 2-3x/month
- ○ 1x/week
- ○ 2x/week
- ○ 3-4x/week
- ○ 5-6x/week
- ○ 1x/day
- ○ 2 or more times/day

During the ***past month***, how often did you eat any processed meat, such as bacon, lunch meats, or hot dogs? Include processed meats you had in sandwiches, soups, pizza, casseroles, and other mixtures. Processed meats are those preserved by smoking, curing or salting or by

the addition of preservatives. Examples are: ham, bacon, pastrami, salami, sausages, bratwursts, frankfurters, hot dogs or spam. Check one.

- Never
- 1x/month
- 2-3x/month
- 1x/week
- 2x/week
- 3-4x/week
- 5-6x/week
- 1x/day
- 2 or more times/day

During the ***past month***, how often did you eat poultry such as chicken or turkey? Include poultry you had in sandwiches, lasagna, stew, and other mixtures. Check one.

- Never
- 1x/month
- 2-3x/month
- 1x/week
- 2x/week
- 3-4x/week
- 5-6x/week
- 1x/day
- 2 or more times/day

During the ***past month***, how often did you eat fish or seafood? Check one.

- ◯ Never
- ◯ 1x/month
- ◯ 2-3x/month
- ◯ 1x/week
- ◯ 2x/week
- ◯ 3-4x/week
- ◯ 5-6x/week
- ◯ 1x/day
- ◯ 2 or more times/day

During the ***past month***, how often did you eat vegetarian meat alternatives and soy products such as tofu, vegetarian sausage, vegetarian sandwich meats etc.? Check one.

- ◯ Never
- ◯ 1x/month
- ◯ 2-3x/month
- ◯ 1x/week
- ◯ 2x/week
- ◯ 3-4x/week
- ◯ 5-6x/week
- ◯ 1x/day
- ◯ 2 or more times/day

During the ***past month***, how often did you eat whole grain bread including toast, rolls, and in sandwiches? Whole grain breads include whole wheat, rye, oatmeal and pumpernickel. Do <u>not</u> include white bread or multigrain bread. Check one.

- ○ Never
- ○ 1x/month
- ○ 2-3x/month
- ○ 1x/week
- ○ 2x/week
- ○ 3-4x/week
- ○ 5-6x/week
- ○ 1x/day
- ○ 2 or more times/day

During the ***past month***, how often did you eat chocolate or any other types of candy? Do <u>not</u> include sugar-free candy. Check one.

- ◯ Never
- ◯ 1x/month
- ◯ 2-3x/month
- ◯ 1x/week
- ◯ 2x/week
- ◯ 3-4x/week
- ◯ 5-6x/week
- ◯ 1x/day
- ◯ 2 or more times/day

During the ***past month***, how often did you eat doughnuts, sweet rolls, danish, muffins, pan dulce, or pop tarts? Do <u>not</u> include sugar-free items. Check one.

- ◯ Never
- ◯ 1x/month
- ◯ 2-3x/month
- ◯ 1x/week
- ◯ 2x/week
- ◯ 3-4x/week
- ◯ 5-6x/week
- ◯ 1x/day
- ◯ 2 or more times/day

During the ***past month***, how often did you eat cookies, cake, pie or brownies? Do <u>not</u> include sugar-free kinds. Check one.

- ○ Never
- ○ 1x/month
- ○ 2-3x/month
- ○ 1x/week
- ○ 2x/week
- ○ 3-4x/week
- ○ 5-6x/week
- ○ 1x/day
- ○ 2 or more times/day

During the ***past month***, how often did you eat ice cream or other frozen desserts? Do <u>not</u> include sugar-free kinds. Check one.

- ◯ Never
- ◯ 1x/month
- ◯ 2-3x/month
- ◯ 1x/week
- ◯ 2x/week
- ◯ 3-4x/week
- ◯ 5-6x/week
- ◯ 1x/day
- ◯ 2 or more times/day

During the ***past month***, did you take any of the following supplements? Check all that apply.

- ☐ Multivitamin
- ☐ Calcium
- ☐ Vitamin D
- ☐ Vitamin B12
- ☐ Vitamin C
- ☐ Iron
- ☐ Omega-3
- ☐ Other, please specify: ______________________________
- ☐ I did not take any supplements

Please specify how often (per week) and how much (units/dosage per intake) of {automatic entry from previous question} you took during the past month:

____

Do you identify as one of the following? Check one.

- ◯ Pescetarian (you eat fish, eggs, dairy, but no meat or poultry)
- ◯ Vegetarian (you eat eggs and dairy, but no fish, meat or poultry)
- ◯ Vegan (you don't eat any animal products)
- ◯ Other, please specify: ______________________________
- ◯ I don't identify as any of the above
- ◯ Prefer not to answer

How old were you when you first identified as {automatic entry from previous question}?

____

What is your reason to identify as {automatic entry from previous question }? Check the option that is your most important reason.

- ◯ Weight loss
- ◯ Health reasons
- ◯ Ethical reasons (i.e. to reduce the suffering of animals)
- ◯ Environmental reasons
- ◯ Religious or cultural reasons
- ◯ Other, please specify: ______________________________
- ◯ Prefer not to answer

Do you have any food allergies? Check all that apply.

- ☐ Peanuts
- ☐ Tree nuts (such as almonds, Brazil nuts, cashews, hazelnuts, macadamias, pecans, pine nuts. pistachios, walnuts)
- ☐ Eggs
- ☐ Lactose
- ☐ Wheat
- ☐ Gluten
- ☐ Sesame
- ☐ Soy
- ☐ Mustard
- ☐ Fish
- ☐ Shellfish (i.e. crustaceans and mollusks)
- ☐ Other, please specify: ____
- ☐ I have no food allergies
- ☐ Prefer not to answer

Awesome, part two is done, too!

In this final and short part of the survey, we will ask you a few questions about yourself. Remember that answering these questions is voluntary. If you choose not to answer a certain question, please check the box “prefer not to say” or leave the text box blank in order to move on to the next question.

How old are you (in years)?

____

Which term do you use to describe your gender identity?

- ○ Woman
- ○ Man
- ○ Trans Woman
- ○ Trans Man
- ○ Gender queer
- ○ Other, please specify: ____
- ○ Prefer not to say

What term best describes your sexual orientation?

- ◯ Asexual
- ◯ Bisexual
- ◯ Gay
- ◯ Lesbian
- ◯ Pan sexual
- ◯ Queer
- ◯ Same gender loving
- ◯ Straight/heterosexual
- ◯ Other, please specify: ____
- ◯ Prefer not to say

What is your relationship status?

- ◯ Not in a relationship
- ◯ In a relationship but not living together
- ◯ In a relationship and living together
- ◯ I'm not sure
- ◯ Prefer not to say

What is your height (please indicate feet and inches or centimeters)?

____

What is your weight (please indicate pounds or kilograms)?

____

How would you describe your ethnic or cultural background?

- ○ Aboriginal/First Nation
- ○ Arab
- ○ Black
- ○ Chinese
- ○ Filipino
- ○ Japanese
- ○ Korean
- ○ Latin American
- ○ South Asian (e.g. Indian, Pakistani, Sri Lankan)
- ○ Southeast Asian (e.g. Vietnamese, Cambodian, Malaysian)
- ○ West Asian (e.g. Iranian, Afghan)
- ○ White
- ○ Other, please specify: ________________________________
- ○ Prefer not to say

Are you an international student?

- ○ Yes
- ○ No
- ○ Prefer not to say

Is English your first language?

- ○ Yes
- ○ No
- ○ Prefer not to say

Where do you currently live?

- ○ Campus residency hall, please specify which one: ________________
- ○ Fraternity or sorority house
- ○ Other university housing, please specify: ________________
- ○ Parent's or guardian's home
- ○ In a house/apartment by myself
- ○ In a house/apartment with room mates
- ○ Other off-campus housing, please specify: ________________
- ○ Prefer not to say

What is your current year in school?

- ○ 1st year undergrad
- ○ 2nd year undergrad
- ○ 3rd year undergrad
- ○ 4th year undergrad
- ○ Higher than 4th year undergrad
- ○ Graduate or professional
- ○ Not seeking a degree
- ○ Other, please specify: ____
- ○ Prefer not to say

What is your field of study?

____

End of Block: Main Survey
