# Supplementary material for: The association between diet and mental health and wellbeing in young adults within a biopsychosocial framework
Source: PLoS One. 2021 Jun 3;16(6):e0252358. doi: 10.1371/journal.pone.0252358 (PMC8174719; doi:10.1371/journal.pone.0252358)
Supplement: Attachment — Submitted filename: PONE-D-20-39899_reviewer #2 de-identified.pdf [file pone.0252358.s002.pdf]

PLOS ONE

The association between diet and mental health and wellbeing in young adults within a biopsychosocial framework

--Manuscript Draft--

**Manuscript Number:**	PONE-D-20-39899
**Article Type:**	Research Article
**Full Title:**	The association between diet and mental health and wellbeing in young adults within a biopsychosocial framework
**Short Title:**	Diet and mental health in a biopsychosocial framework
**Corresponding Author:**	Verena Rossa-Roccor, MD, MSc<br>The University of British Columbia<br>Vancouver, CANADA
**Keywords:**	Depression; anxiety; quality of life; dietary patterns; plant-based diets; planetary health
**Abstract:**	Objective: Predominantly plant-based diets can co-benefit human physical health and the planet. Young adults appear to be on the forefront of the shift to plant-based diets. However, little is known about the relationship between plant-based diets and mental health in this population even though mental health disorders contribute substantially to the global burden of disease, particularly among this age group.<br>Design: In this cross-sectional study we utilize a biopsychosocial framework to assess the association between dietary intake and mental health and wellbeing. Mental health was assessed using self-reported measures of anxiety (GAD-7), depression (PHQ-9) and quality of life (single-item). Dietary intake in the prior month was assessed using a dietary screener (DSQ) and participants were asked to self-identify a diet preference (e.g., vegan).<br>Setting and participants: 339 university undergraduate students.<br>Results: A principle component analysis of dietary intake found three dominant dietary patterns (plant-based, animal-based, and 'junk foods'); 28.1% (n=95) of participants self-identified as pescatarian, vegetarian, vegan, other. The association between dietary patterns, diet preference and mental health was assessed through regression analysis. After controlling for covariables, we found a significant positive association between the junk food component and depression (z-score β=.21, p≤.001; adj. R2=.39) and anxiety (z-score β=.14; p≤.001; adj. R2=.32) while no association was found between plant-based, animal-based or self-identified diet preference and the mental health measures.<br>Conclusions: Predominantly plant-based diet patterns are not negatively associated with mental health and wellbeing. It is important to consider dietary composition and to conceptualize diet as a health behaviour that is embedded in a biopsychosocial framework.
**Order of Authors:**	Verena Rossa-Roccor, MD, MSc
	Chris G Richardson
	Rachel A Murphy
	Anne M Gadermann
**Additional Information:**	
**Question**	**Response**
**Financial Disclosure**<br><br>Enter a financial disclosure statement that describes the sources of funding for the work included in this submission. Review the submission guidelines for detailed requirements. View published research	This research did not receive any specific grant from funding agencies in the public, commercial, or not-for-profit sectors. VR-R was the recipient of a Canadian Institutes of Health Research Canada Graduate Scholarships - Master's award during the duration of the study. RAM is supported by the Canadian Cancer Society (grant #704735) and the Michael Smith Foundation for Health Research (grant #17644). AMG gratefully acknowledges support from the Michael Smith Foundation for Health Research (grant # 17717). The funders had no role in study design, data collection and analysis,

Powered by Editorial Manager® and ProduXion Manager® from Aries Systems Corporation

articles from *PLOS ONE* for specific examples.<br><br>This statement is required for submission and **will appear in the published article** if the submission is accepted. Please make sure it is accurate.<br><br>**Unfunded studies**<br>Enter: *The author(s) received no specific funding for this work.*<br><br>**Funded studies**<br>Enter a statement with the following details:<br>• Initials of the authors who received each award<br>• Grant numbers awarded to each author<br>• The full name of each funder<br>• URL of each funder website<br>• Did the sponsors or funders play any role in the study design, data collection and analysis, decision to publish, or preparation of the manuscript?<br>• **NO** - Include this sentence at the end of your statement: *The funders had no role in study design, data collection and analysis, decision to publish, or preparation of the manuscript.*<br>• **YES** - Specify the role(s) played.<br><br>* typeset	decision to publish, or preparation of the manuscript.
**Competing Interests**<br><br>Use the instructions below to enter a competing interest statement for this submission. On behalf of all authors, disclose any competing interests that could be perceived to bias this work—acknowledging all financial support and any other relevant financial or non-financial competing interests.<br><br>This statement **will appear in the published article** if the submission is accepted. Please make sure it is accurate. View published research articles from *PLOS ONE* for specific examples.	I have read the journal's policy and the authors of this manuscript have the following competing interests:The authors declare that they have no conflict of interest. RAM has received funds as a consultant from Pharmavite and research funds from the International Life Sciences Institute, North America. Neither relationship is relevant to the work presented in this manuscript.

**NO authors have competing interests**<br><br>Enter: *The authors have declared that no competing interests exist.*<br><br>**Authors with competing interests**<br><br>Enter competing interest details beginning with this statement:<br><br>*I have read the journal's policy and the authors of this manuscript have the following competing interests: [insert competing interests here]*<br><br>* typeset	
**Ethics Statement**<br><br>Enter an ethics statement for this submission. This statement is required if the study involved:<br><br>• Human participants<br>• Human specimens or tissue<br>• Vertebrate animals or cephalopods<br>• Vertebrate embryos or tissues<br>• Field research<br><br>Write "N/A" if the submission does not require an ethics statement.<br><br>General guidance is provided below. Consult the submission guidelines for detailed instructions. **Make sure that all information entered here is included in the Methods section of the manuscript.**	This study was conducted according to the guidelines laid down in the Declaration of Helsinki and all procedures involving research study participants were approved by the University of British Columbia Behavioural Research Ethics Board (review code H18-00442). Written informed consent was obtained from all participants.

Powered by Editorial Manager® and ProduXion Manager® from Aries Systems Corporation

**Format for specific study types**<br><br>**Human Subject Research (involving human participants and/or tissue)**<br>• Give the name of the institutional review board or ethics committee that approved the study<br>• Include the approval number and/or a statement indicating approval of this research<br>• Indicate the form of consent obtained (written/oral) or the reason that consent was not obtained (e.g. the data were analyzed anonymously)<br><br>**Animal Research (involving vertebrate animals, embryos or tissues)**<br>• Provide the name of the Institutional Animal Care and Use Committee (IACUC) or other relevant ethics board that reviewed the study protocol, and indicate whether they approved this research or granted a formal waiver of ethical approval<br>• Include an approval number if one was obtained<br>• If the study involved *non-human primates*, add *additional details* about animal welfare and steps taken to ameliorate suffering<br>• If anesthesia, euthanasia, or any kind of animal sacrifice is part of the study, include briefly which substances and/or methods were applied<br><br>**Field Research**<br><br>Include the following details if this study involves the collection of plant, animal, or other materials from a natural setting:<br>• Field permit number<br>• Name of the institution or relevant body that granted permission	
**Data Availability**<br><br>Authors are required to make all data underlying the findings described fully available, without restriction, and from the time of publication. PLOS allows rare exceptions to address legal and ethical concerns. See the PLOS Data Policy and FAQ for detailed information.	Yes - all data are fully available without restriction

Powered by Editorial Manager® and ProduXion Manager® from Aries Systems Corporation

A Data Availability Statement describing where the data can be found is required at submission. Your answers to this question constitute the Data Availability Statement and **will be published in the article**, if accepted.<br><br>**Important:** Stating 'data available on request from the author' is not sufficient. If your data are only available upon request, select 'No' for the first question and explain your exceptional situation in the text box.<br><br>Do the authors confirm that all data underlying the findings described in their manuscript are fully available without restriction?	
**Describe where the data may be found in full sentences. If you are copying our sample text, replace any instances of XXX with the appropriate details.**<br><br>• If the data are **held or will be held in a public repository**, include URLs, accession numbers or DOIs. If this information will only be available after acceptance, indicate this by ticking the box below. For example: *All XXX files are available from the XXX database (accession number(s) XXX, XXX.).*<br>• If the data are all contained **within the manuscript and/or Supporting Information files**, enter the following: *All relevant data are within the manuscript and its Supporting Information files.*<br>• If neither of these applies but you are able to provide **details of access elsewhere**, with or without limitations, please do so. For example:<br><br>*Data cannot be shared publicly because of [XXX]. Data are available from the XXX Institutional Data Access / Ethics Committee (contact via XXX) for researchers who meet the criteria for access to confidential data.*<br><br>*The data underlying the results presented in the study are available from (include the name of the third party*	All relevant data are within the manuscript and its Supporting Information files

and contact information or URL).<br>• This text is appropriate if the data are owned by a third party and authors do not have permission to share the data.<br><br>* typeset	
Additional data availability information:	

Powered by Editorial Manager® and ProduXion Manager® from Aries Systems Corporation

Manuscript

Click here to access/download;Manuscript;Manuscript_PLOS One_final.docx

Full title: The association between diet and mental health and wellbeing in young adults within a biopsychosocial framework

Short title: Diet and mental health in a biopsychosocial framework

Verena Rossa-Roccor[1,2], Chris G. Richardson[1,3], Rachel A. Murphy[1,5], Anne M. Gadermann[1,3,4]

[1] School of Population and Public Health, University of British Columbia, 2206 East Mall, Vancouver, BC, V6T1Z3, Canada

[2] Institute for Resources, Environment and Sustainability, University of British Columbia, 429-2202 Main Mall, Vancouver, BC, V6T1Z4

[3] Centre for Health Evaluation and Outcome Sciences, Providence Health Care Research Institute, 588-1081 Burrard Street, St. Paul's Hospital, Vancouver, BC, V6Z1Y6, Canada

[4] Human Early Learning Partnership, School of Population and Public Health, University of British Columbia, Suite 440, 2206 East Mall, Vancouver, BC, V6T1Z3, Canada

[5] Cancer Control Research, BC Cancer, 2-107 675 W 10th Ave, Vancouver, BC, V5Z1L3, Canada

Corresponding author:

Email: verena.rossa-roccor@ubc.ca [VR-R]

Abstract

Objective: Predominantly plant-based diets can co-benefit human physical health and the planet. Young adults appear to be on the forefront of the shift to plant-based diets. However, little is known about the relationship between plant-based diets and mental health in this population even though mental health disorders contribute substantially to the global burden of disease, particularly among this age group.

Design: In this cross-sectional study we utilize a biopsychosocial framework to assess the association between dietary intake and mental health and wellbeing. Mental health was assessed using self-reported measures of anxiety (GAD-7), depression (PHQ-9) and quality of life (single-item). Dietary intake in the prior month was assessed using a dietary screener (DSQ) and participants were asked to self-identify a diet preference (e.g., vegan).

Setting and participants: 339 university undergraduate students.

Results: A principle component analysis of dietary intake found three dominant dietary patterns (plant-based, animal-based, and 'junk foods'); 28.1% (n=95) of participants self-identified as pescatarian, vegetarian, vegan, other. The association between dietary patterns, diet preference and mental health was assessed through regression analysis. After controlling for covariables, we found a significant positive association between the junk food component and depression (z-score β=.21, $p \leq .001$; adj. R^2=.39) and anxiety (z-score β=.14; $p \leq .001$; adj. R^2=.32) while no association was found between plant-based, animal-based or self-identified diet preference and the mental health measures.

Conclusions: Predominantly plant-based diet patterns are not negatively associated with mental health and wellbeing. It is important to consider dietary composition and to conceptualize diet as a health behaviour that is embedded in a biopsychosocial framework.

1. Introduction

Holistic health fields of enquiry such as planetary health view diet as being embedded in a complex system of interrelations between individual, social, cultural, and environmental factors. In March 2019, the EAT Lancet Commission on Healthy Diets from Sustainable Food Systems put forward the first global benchmark diet capable of sustaining human and planetary health [1]. The recommendations herein include two sets of frameworks: on the one hand, they specify food intake ensuring human health and on the other hand, they suggest specific planetary boundaries for food production. More specifically, these 'win-win diets' highlight the co-benefits of plant- over animal-based foods. The authors state that "when viewed together as an integrated human health and environmental sustainability agenda, 'win-win' diets, that fall within the safe operating space for food systems, will help to achieve global human health and environmental sustainability goals" [1].

The authors of this landmark report were able to draw on extensive evidence on the benefit of planetary healthy, i.e., predominantly plant-based diets, on physical health. However, the potential mental health impacts of these diets remain largely unknown. This lack of evidence is surprising given that mental and behavioural disorders are the leading cause of years lived with disability worldwide [2]. Depression and anxiety are the two leading mental health disorders in terms of global disease burden: Depressive disorders account for 40% of disability-adjusted life years (DALYs), anxiety disorders account for 15% of DALYs caused by all mental and substance use disorders [2]. Lifetime prevalence rates range from 10 to 15% for depression [3] and average 17% for all anxiety disorders combined [4]. Furthermore, mental illness often develops into a chronic, lifelong health issue that can have profound and devastating effects on

an individual's life trajectory by impacting and disrupting social functioning and capital [5], educational attainment [6], economic output [7], and overall quality of life (QoL) [8].

Approximately 75% of all mental illnesses have their onset before the age of 25 [9–11]. University students in particular are vulnerable for depression, anxiety, and substance use disorders [12] and mental health issues within this population are on the rise [13,14]. This warrants interventions that support students in this developmental phase which offers potential for preventive and early intervention as it is accompanied by significant brain development with elevated neural plasticity [15,16]. To meet this growing need, research in the area of nutritional psychiatry is now considering dietary interventions to prevent and treat mental illnesses [17]. These interventions have the potential to contribute to improved emotional functioning and long-term health, as the adoption of a healthy diet during this developmental period may contribute substantially to the prevention of (chronic) non-communicable diseases in later stages of life [18].

Young people are also particularly likely to adopt plant-based diets [19,20]. Current estimates see approximately 7% of Canada's population self-identifying as vegetarian or vegan (compared to only 2% in 2003) – with those under the age of 35 being three times more likely than older generations to identify as vegetarian or vegan while predictions see this number increasing rapidly [20]. The numbers of those who do not completely abstain from meat or other animal-based products but aim to substantially decrease their consumption, particularly of greenhouse gas and water-intense red meats and ruminant products, are even higher: According to recent consumer polls, 43% of Canadians are aiming to incorporate more plant-based foods into their diets [21] which is reflected in a constant decline of overall per capita meat consumption in Canada over the last three decades [22].

There are different definitions and conceptualizations of plant-based diets. One approach is to assess diet preference, i.e., someone identifies as vegetarian, vegan, or newer categories such as ‘flexitarian’, a term describing individuals who eat “primarily vegetarian with the occasional inclusion of meat or fish” [23]. Using this categorical definition of plant-based diets, preliminary findings ranged from vegetarians reporting significantly better mood and less anxiety and stress compared to non-vegetarians [24,25] to vegetarians having higher odds of lifetime prevalence of depression, anxiety, and physical disorders compared to non-vegetarians [26–30]. However, findings show that self-report of diet preference (i.e., stating whether one identifies as vegan, vegetarian, etc.) says very little about actual diet pattern and quality [31]. Certain plant-based foods such as whole grains, vegetables, legumes, nuts, and fruits are indeed known to have health benefits while high intake of others such as refined grains, fried potatoes, sweets and desserts, or fruit juices are generally considered unhealthy [32]. Therefore, compositions of diet patterns and diet quality of those who describe themselves as vegetarians, vegans, pescatarians, etc. likely differ greatly between individuals and need to be assessed more carefully. Research on the association between diet and mental health utilizing composite dietary measures such as dietary patterns and diet quality indices also shows heterogenous results. However, the trend seems to point towards better mental health among those following predominantly plant-based diets, i.e., diets that are high in vegetable, fruit, whole grain intake with moderate intake of fish and worse mental health among those following a ‘Western’ diet high in animal and processed foods [33–35].

In this study, we conceptualized plant-based diets as diet patterns that consist mostly or exclusively of plant-based foods. We assessed diet through two approaches: using a categorical definition of plant-based diet asking respondents to self-identify according to diet preferences

(no preference, pescatarian, vegetarian, vegan, other with open text entry option) and the diet pattern-based approach through dietary pattern analysis. We then compared both approaches in terms of their association with depression, anxiety, and QoL hypothesizing that diet patterns high in plant foods rather than diet preference would be negatively associated with the outcomes.

One limitation that all previous studies on this topic have in common is their narrow focus on a primarily biomedical understanding of the relationship between diet and mental health. Neither mental health nor dietary behaviours exist in a vacuum. As described in an extensive body of research, stress, stressful life events, body image, physical activity, sleep, and social support are all predictors for mental health and wellbeing outcomes [36–41]. Simultaneously, these factors are conceptually related to diet and therefore fulfill the criteria of presenting possible confounders in the relationship under investigation in this study [42–47]. Previous studies have not sufficiently considered these factors, particularly the social dimension of dietary habits, in their theoretical frameworks and statistical models.

With this study, we therefore sought to address several gaps in the literature. We assessed diet patterns in a population of undergraduate university students. We further examined whether plant-based diet pattern and self-reported preferences are associated with mental health (depression and anxiety) and wellbeing (QoL) in this population (for simplicity, we refer to both as ‘mental health’ herein). Finally, we extend the understanding of this relationship by considering this question within a biopsychosocial rather than a currently predominant biomedical framework in this field, thereby adding important confounding variables to the analysis.

2. Methods

2.1. Study design and participants

The study design was cross-sectional. We collected data through an online self-report survey from March to April of 2019. The main outcome variables of interest were depression, anxiety, and QoL, as indicator of overall mental wellbeing. The main explanatory variable was diet as assessed through dietary pattern over the prior month as well as self-reported diet preference. The survey contained additional items on social support, health behaviours and status, body image, stress, stressful life events, and socioeconomic background.

We recruited participants among undergraduate students at the University of British Columbia (UBC), Vancouver, Canada through convenience sampling; data was collected anonymously. Excluding graduate students (n=9) and cases that were missing items for any of the main outcome or main explanatory variables (n=92) yielded a final analytic sample of n=339 respondents.

2.2. Measures

To assess dietary habits, we used the U.S. National Cancer Institute's Dietary Screening Questionnaire (DSQ) which asks about the frequency of consumption of select foods and beverages in the past 30 days. Evaluations have shown good agreement between estimates of intakes between the DSQ and multiple 24hr recalls with differences in means <2% and differences in prevalence <16% [48]. In its original version, the DSQ includes 26 items. The questionnaire was slightly altered in order to make it more appropriate for the local context and to include items that were relevant to this study such as consumption of poultry, additional dairy

products, vegetarian meat alternatives, and non-dairy milk. The final version used in this study had 28 items (see supplementary materials for questionnaire).

In addition to the DSQ, we included one item asking about dietary preference. Participants were asked if they identified as: a) pescatarian (‘you eat fish, eggs, and dairy but no meat or poultry’); b) vegetarian (‘you eat eggs and dairy but no fish, meat or poultry’); c) vegan (‘you don’t eat any animal products’); d) other (‘please specify’; participants were given the option to enter text); e) none of the above.

We assessed QoL as a measure for overall mental wellbeing through a single-item measure (“In general, would you say your quality of life is…”) with responses rated on a 5-point Likert scale (0=poor, 1=fair, 2=good, 3=very good, 4=excellent). This single-item measure is one of the most widely used items to measure QoL and has been included in routinely used assessment tools such as the Patient-Reported Outcomes Measurement Information System Scale version 1.2 PROMIS® [49].

We assessed depressive symptoms using the 9-item Patient Health Questionnaire (PHQ-9) which is widely used in both clinical and research settings and has been validated for a variety of populations to detect and assess severity of depressive symptoms [50–53]. The total score ranges from 0 to 27. PHQ-9 scores of ≥10 have been reported to have a sensitivity of 88% and a specificity of 88% for major depression [53]. For clinical and diagnostic purposes, the measure can further be used to assess severity of symptoms applying cut-off scores. Cut-off scores for mild, moderate, moderately severe, and severe depression were found to be 5, 10, 15, and 20, respectively [53]. In general, a score ≥10 means that further clinical evaluation is indicated while a score ≥20 indicates that the individual may require psychotherapy and/or medication.

We assessed anxiety symptoms using the 7-item General Anxiety Disorder Questionnaire (GAD-7). Similar to the PHQ-9, this is a standard instrument to detect and assess the severity of anxiety disorder used widely for both clinical and research practices. Although originally designed to detect general anxiety disorder, it has been found that the GAD-7 is useful as a screening instrument for related anxiety disorders such as post-traumatic stress disorder, social anxiety disorder, and panic disorder [54]. The total score ranges from 0 to 21; for GAD-7 scores ≥10, sensitivity and specificity have been reported to be above 80% [55]. Much like the PHQ-9, the GAD-7 can further be used to assess severity of symptoms by applying cut-off scores. Cut-off scores for mild, moderate, and severe anxiety were found to be 5, 10, and 15, respectively [55]. In general, a score ≥10 means that further clinical evaluation is indicated while a score ≥15 indicates that the individual may require psychotherapy and/or medication.

2.3. Statistical analyses and missing data

For descriptive purposes, we reported continuous variables through the mean and standard deviation (SD); for categorical variables, we reported frequencies.

The final sample consisted of n=339 participants. In this analytic sample, the data on the main variables of interest (QoL, depression, anxiety, DSQ) was complete for all respondents. For covariables, responses such as ‘prefer not to say’ and ‘don’t know’ were treated as missing data. We applied multiple imputation (Markov Chain Monte Carlo Method; five imputations) to address missing data for all covariables that were to be included in the multiple regression model based on the conceptual understanding of the relationship between diet and mental health in order to avoid underestimation of sampling error [56].

We applied principal component analysis (PCA) with varimax rotation as a data reduction approach for the evaluation of the DSQ items. The decision on how many components were to be retained was based on considering the combination of interpretability and conceptual reasoning of the emerging components, the eigenvalues (>1), the scree plot, and the percentage of variance explained by the components. Varimax rotation was chosen as it was assumed that emerging components would not be highly correlated with each other.

The PCA component scores for each participant were entered into regression models as the main explanatory variable when examining the relationship between diet pattern and mental health outcomes (using the total scores of the PHQ-9 and GAD-7 measures). We built three nested linear regression models per outcome using a hierarchical approach. We first entered sociodemographic factors (age, gender, ethnicity), then added lifestyle-related variables (physical activity, sleep, weight satisfaction, stress, stressful life events). The third step was to add social support as a known individual predictor for mental health. Finally, we added the main variables of interest (PCA component scores) to assess its additional contribution to the outcome of interest. These nested models were built for each outcome variable of interest: Model 1: QoL; Model 2: Depression; Model 3: Anxiety. This approach was repeated with self-reported diet preference as the main explanatory variable. Assumptions for linear models were met. All analyses were 2-tailed with a significance level of $p \leq 0.05$ and conducted with IBM SPSS Statistics 25®.

3. Results

3.1. Sample characteristics and covariates

The total sample consists of n=339 participant[illegible]**able 1** depicts detailed sample characteristics as well as frequencies of covariables that were included in the regression models such as health behaviours (physical activity and sleep), body image, overall stress, stressful life events, and social support. Overall, we found that almost none of the students (96.1%, n=326) met the recommended amount of moderate or vigorous physical activity in the previous week. Three quarters of the participants (76.7%, n=260) reported enough sleep to feel rested on a maximum of four days in the previous week. Two thirds of the students (66.6%, n=226) experienced more than average or even tremendous stress over the 12 months preceding the survey. Approximately half of the students were somewhat, very, or extremely satisfied with their weight (52.6%, n=178). Experiencing stressful life events that caused moderate or severe stress was reported by 76.3% (n=259) of the students. Conversely, the majority of participants (80.4%, n=272) reported having good, very good, or excellent satisfaction with their social relationships and activities.

Table 1 Participant demographic and psychosocial characteristics

Characteristic/Item	Item categories	mean	SD	n[†]	%
Age		19.5	1.9		
Gender identity	Female			224	66.1
	Male			109	32.1
	Other (trans, queer, other)			6	1.8
Sexual orientation	Heterosexual			257	75.8
	Bisexual			33	9.7
	Gay/Lesbian			6	1.8
	Other			28	8.3
Relationship status	Not in a relationship			221	65.2
	In a relationship			95	28.0
	Not sure			12	3.5

Characteristic/Item	Item categories	mean	SD	n†	%
Ethnicity	White			156	46.0
	Asian			135	39.8
	Other			48	14.2
Year in school	1st year			211	62.2
	2nd year			64	18.9
	3rd year			28	8.3
	4th year			19	5.6
	Higher than 4th year undergrad			9	2.7
	Not seeking a degree			1	0.3
International student	Yes			120	35.4
	No			213	62.8
Residence	On-campus			248	73.2
	With parents			34	10.0
	Off-campus alone/with roommates/other			48	14.2
Physical activity in the past 7 days *(20min of vigorous exercise or 30min of moderate exercise)*	Never			94	27.6
	1-3 days/week			167	49.3
	4-6 days/week			65	19.1
	every day or more than once a day			13	3.9
Enough sleep to feel rested in the morning in the past 7 days	≤ 4 days/week			260	76.7
	≥ 5 days/week			79	23.1
Weight satisfaction	not satisfied/slightly unsatisfied			161	47.4
	somewhat satisfied			101	29.9
	very/extremely satisfied			77	22.7
Perceived stress	no/less than average stress			25	7.4
	average stress			88	26.0
	more than average/tremendous stress			226	66.6
Stressful life events	mild stressors			81	23.8
	moderate stressors			149	44.1
	severe stressors			109	32.2
Social support	poor/fair			67	19.6
	good/very good/excellent			272	80.4

†n may vary due to missing data

3.2. Diet

Three dietary components emerged from the PCA of the DSQ items. Component 1 (plant foods) was high in plant-based foods and non-animal-based dairy and meat alternatives as well

as whole grains. Component 2 (animal foods) was high in animal-based foods such as different meats and dairy products. Component 3 (junk foods) was high in processed foods, snacks, and candies. The total variance explained by the retained three components was 40.6%. Details on loadings per component for each food item/group after varimax rotation can be seen in **Table 2**. For better interpretability, we removed food items/groups that did not load ≥0.4 on either of the components (namely, potatoes, tomato sauce, and fruit juice) from the final analysis [57]. In addition, loadings below 0.4 are omitted from the table to improve readability.

Table 2 Principal component analysis of dietary components and component loadings for dietary patterns after varimax rotation

**Food item/group**	**Component 1 (plant foods)**	**Component 2 (animal foods)**	**Component 3 (junk foods)**
Brown rice and whole grains	0.70		
Beans and legumes	0.68		
Nuts and seeds	0.66		
Green leafy vegetables	0.66		
Other vegetables	0.64		
Fruit	0.63		
Vegetarian/vegan meat alternatives	0.53	-0.46	
Non-dairy milk	0.51	-0.41	
Whole grain bread	0.49		
Cereal	0.43		
Poultry		0.80	
Red meat		0.75	
Processed meat		0.68	
Fish and seafood		0.61	
Cheese		0.56	
Yoghurt		0.50	
Dairy milk		0.49	
Cookies, cake, pie			0.65
Ice cream			0.61
Donuts etc.†			0.60
Chocolate and candy			0.60
Soda			0.54
Pizza			0.51
Fried potatoes			0.50
Coffee or tea with sugar			0.41

†this item on the questionnaire included donuts, sweet rolls, Danish, muffins, pan dulce, and pop-tarts

Almost one third of students (28.1%, n= 95) self-identified as either pescatarian, vegetarian, vegan or other (which were mostly on a spectrum of non-mainstream preferences such as reducetarian or flexitarian). See **Table 3** for details.

Table 3 Diet preference

Diet preference	n	%
Pescatarian	13	4.0
Vegetarian	19	5.5
Vegan	37	10.8
Other	26	7.8
Do not identify as any of the above	244	71.9

3.3. Mental health and wellbeing

As can be seen in **Table 4**, more than half of the participants (56.3%, n=193) reported their overall QoL to be either very good or excellent with a mean score of 2.6 (±1.0) out of 5. The mean score for depression was 9.3 (±6.1) out of 27; the mean score for anxiety was 7.9 (±5.8) out of 21. In terms of clinical relevance, 75% (n=254) of students had scores which indicate the need for further evaluation concerning symptoms of depression; for anxiety 65.1% (n=221) had scores indicating need for further evaluation. Of those who scored above 10 points for depression (n=142), 16% (n=23) would likely benefit from psychotherapy and/or medication; for those who scored above 10 points for anxiety (n=110) this proportion is even higher with 48% (n=53).

Table 4 Mental health and wellbeing

**Mental health item**	**Item categories**	**mean**	**SD**	**n**	**%**
QoL continuous (0 to 5)		2.6	1.0		
QoL ordinal	Poor			10	2.8
	Fair			31	9.2
	Good			105	31.1
	very good			138	40.1
	Excellent			55	16.2
Depression score (0 to 27)		9.3	6.1		
Depression severity	no depression			85	25.0
	mild depression†			112	32.9
	moderate depression			73	21.7
	moderately severe depression			46	13.6
	severe depression‡			23	6.8
Anxiety score (0 to 21)		7.9	5.8		
Anxiety severity	no anxiety			118	34.8
	mild anxiety			111	32.7
	moderate anxiety			57	16.8
	severe anxiety‡			53	15.6

Abbreviation: QoL, quality of life
†Cut-off for further evaluation
‡Psychotherapy and/or medication are indicated

3.4. Association between diet and mental health and wellbeing

The unadjusted linear regression analysis shows a significant association between several variables. The plant food dietary component was positively associated with QoL (β=.20, $p\leq$.001). The junk food component was positively associated with depression (β=.26, $p\leq$.001), while the animal food component and the plant food component were negatively associated with depression (β=-.07, $p\leq$.05 and β=-.10, $p\leq$.001, respectively). The junk food component was further positively associated with anxiety (β=.18, p=.001) and the animal food component was negatively associated with anxiety (β=-.09, $p\leq$.001). After adjusting for all covariables, the positive associations between the junk food component and depression and anxiety remain significant.

Model 1 (dietary pattern and QoL): After adjusting for all covariables, statistically significant negative associations were found between Asian ethnicity, stress, and QoL; significant positive associations were found for physical activity, weight satisfaction, and social support with QoL. Social support showed the strongest positive association for QoL (β=.51 increase in QoL score; $p \leq .001$).

Model 2 (dietary pattern and depression): After adjusting for all covariables, statistically significant negative associations were found between sleep, weight satisfaction, and social support with depression; a statistically significant positive association was found for stress and the junk food dietary component (β=.21 increase in depression score; $p \leq .001$; Δadj. R^2=.04).

Model 3 (dietary pattern and anxiety): After adjusting for all covariables, statistically significant positive associations were found between female gender, stress, stressful life events, and the junk food dietary component (β=.14 increase in anxiety score; p=.002; Δ adj. R^2=.01) with anxiety. Social support was significantly negatively associated with anxiety.

Table 5 shows the detailed results for the three hierarchical multiple linear regression models that examined the association between dietary patterns and mental wellbeing outcomes controlling for covariables that reflected a biopsychosocial understanding of the relationship. Δadj. R^2 for each hierarchical step are reported in the footnotes.

Diet preference was not significantly associated with any of the outcome variables. Results are available upon request for these statistically non-significant findings.

Table 5 Unadjusted and adjusted effects of principal component analysis (PCA) diet components on quality of life (QoL), depression, and anxiety

	**Model 1: QoL**			**Model 2: Depression**			**Model 3: Anxiety**		
	**Beta**	**SE Beta**	**Standardized Beta (*β*)**	**Beta**	**SE Beta**	**Standardized Beta (*β*)**	**Beta**	**SE Beta**	**Standardized Beta (*β*)**
Step 1									
**Constant**	3.72	0.52	0.08	3.58	3.36	-0.10	3.31	3.15	-0.25
**Age**	-0.05	0.03	**-0.11***	0.26	0.17	0.09	0.16	0.16	0.05
**Female gender†**	0.20	0.12	0.21	-0.10	0.71	-0.02	1.31	0.67	**0.23***
**Other gender†**	-0.52	0.42	-0.55	4.57	2.80	0.75	4.68	2.62	0.81
**Asian ethnicity‡**	-0.50	0.11	**-0.52****	1.34	0.72	0.22	0.79	0.68	0.14
**Other ethnicity‡**	-0.02	0.16	-0.02	0.73	1.02	0.12	1.40	0.96	0.24
Step 2									
**Constant**	3.55	0.54	-0.03	5.62	3.13	0.05	0.85	3.00	-0.12
**Age**	-0.03	0.03	-0.07	0.08	0.14	0.03	0.02	0.14	0.01
**Female gender†**	0.26	0.10	**0.27***	-0.84	0.60	-0.14	0.63	0.58	0.11
**Other gender†**	-0.11	0.40	-0.11	1.10	2.36	0.18	1.49	2.26	0.26
**Asian ethnicity‡**	-0.40	0.10	**-0.41****	0.56	0.62	0.09	0.35	0.60	0.06
**Other ethnicity‡**	0.07	0.14	0.07	0.04	0.86	0.01	0.76	0.82	0.13
**Sleep**	0.04	0.02	0.09	-0.58	0.15	**-0.20****	-0.32	0.14	**-0.12***
**Physical activity**	0.07	0.02	**0.14***	-0.36	0.15	**-0.12***	-0.19	0.14	-0.07
**Stress**	-0.30	0.06	**-0.27****	2.16	0.34	**0.31****	2.55	0.33	**0.39****
**Stressful life events**	0.02	0.07	0.02	0.72	0.40	0.09	1.02	0.39	**0.13***
**Weight satisfaction**	0.11	0.04	**0.12***	-1.15	0.27	**-0.20****	-0.54	0.26	**-0.10***
Step 3									
**Constant**	1.74	0.48	0.06	10.54	3.24	0.01	4.43	3.13	-0.15
**Age**	-0.01	0.02	-0.01	-0.01	0.14	-0.01	-0.04	0.14	-0.02
**Female gender†**	0.06	0.09	0.06	-0.30	0.60	-0.05	1.02	0.58	0.18
**Other gender†**	-0.22	0.33	-0.23	1.40	2.30	0.23	1.70	2.23	0.30
**Asian ethnicity‡**	-0.28	0.09	**-0.30***	0.26	0.60	0.04	0.13	0.59	0.02
**Other ethnicity‡**	0.11	0.12	0.11	-0.06	0.83	-0.01	0.69	0.81	0.12
**Sleep**	0.01	0.02	0.03	-0.50	0.14	**-0.17****	-0.19	0.14	-0.09
**Physical activity**	0.06	0.02	**0.13***	-0.35	0.14	**-0.11***	-0.19	0.14	-0.07
**Stress**	-0.17	0.05	**-0.16****	1.84	0.34	**0.27****	2.31	0.33	**0.36****
**Stressful life events**	0.05	0.06	0.04	0.65	0.39	0.08	0.96	0.38	**0.13***
**Weight satisfaction**	0.09	0.04	**0.10***	-1.10	0.26	**-0.19****	-0.50	0.26	**-0.10***

	Model 1: QoL			Model 2: Depression			Model 3: Anxiety		
	**Beta**	**SE Beta**	**Standardized Beta (β)**	**Beta**	**SE Beta**	**Standardized Beta (β)**	**Beta**	**SE Beta**	**Standardized Beta (β)**
**Social support**	0.46	0.04	**0.51****	-1.26	0.28	**-0.22****	-0.92	0.27	**-0.17****
Step 4									
**Constant**	1.77	0.49	0.07	10.30	3.22	-0.02	4.20	3.15	-0.17
**Age**	-0.01	0.02	-0.01	0.01	0.14	0.01	-0.04	0.14	-0.01
**Female gender†**	0.04	0.10	0.04	-0.02	0.63	-0.01	1.28	0.62	**0.22***
**Other gender†**	-0.23	0.34	-0.24	1.41	2.26	0.23	1.73	2.22	0.30
**Asian ethnicity‡**	-0.28	0.09	**-0.29***	0.23	0.60	0.04	0.08	0.60	0.01
**Other ethnicity‡**	0.11	0.12	0.12	0.02	0.82	0.01	0.72	0.80	0.12
**Sleep**	0.01	0.02	0.02	-0.49	0.14	**-0.17****	-0.25	0.14	-0.09
**Physical activity**	0.06	0.02	**0.12***	-0.25	0.15	-0.08	-0.10	0.15	-0.04
**Stress**	-0.17	0.05	**-0.16****	1.82	0.33	**0.27****	2.30	0.33	**0.36****
**Stressful life events**	0.05	0.06	0.04	0.42	0.38	0.05	0.81	0.39	**0.11***
**Weight satisfaction**	0.09	0.04	**0.10***	-0.96	0.26	**-0.17****	-0.42	0.25	-0.08
**Social support**	0.46	0.04	**0.51****	-1.36	0.28	**-0.23****	-0.97	0.27	**-0.18****
**PCA plant foods**	0.04	0.04	0.05	-0.07	0.30	-0.01	-0.19	0.29	-0.03
**PCA animal foods**	0.01	0.04	0.01	-0.15	0.28	-0.02	-0.14	0.28	-0.02
**PCA junk foods**	-0.01	0.04	-0.01	1.26	0.27	**0.21****	0.83	0.27	**0.14***

†Reference category: Male gender
‡Reference category: Caucasian ethnicity
**$p \leq .05$*
*** $p \leq .001$.*
Note Model 1: Adjusted R^2 =.08 for Step 1; Δ adj R^2 =.13 for Step 2; Δ adj R^2 =.23 for Step 3; Δ adj R^2 =.00 for Step 4
Note Model 2: Adjusted R^2 =.01 for Step 1; Δ adj R^2 =.30 for Step 2; Δ adj R^2 =.04 for Step 3; Δ adj R^2 =.04 for Step 4
Note Model 3: Adjusted R^2 =.02 for Step 1; Δ adj R^2 =.27 for Step 2; Δ adj R^2 =.02 for Step 3; Δ adj R^2 =.01 for Step 4

4. Discussion

4.1. Interpretation

The final adjusted regression models show that the junk food component score was positively associated with depression and anxiety while there were no significant associations between the plant food or the animal food component and any of the mental health outcomes. While the additional variance explained by the dietary component (junk food) with regard to the mental health outcomes seems small (Δ adjusted R^2 for the model with depression as outcome = .04 and = .01 for the model with anxiety as outcome, respectively), the magnitude of the standardized regression coefficient is comparable to other covariates in the model that are known to be strongly associated with mental health outcomes (e.g., social support). There are two possible explanations for this. In line with the understanding that mental health and diet exist within a biopsychosocial framework, food intake contributes to a complex network of variables that reduce or enhance the risk for adverse mental health outcomes such as social support and relationships. Second, it has been found that self-reported dietary data typically leads to an underestimation of associations [58]. The possibility of underestimation of the association is therefore likely in this study which would mean that the true effect size may be larger. The non-significant trend in the expected direction for the association of the plant food component with mental health outcomes after adjusting for the covariables in this study should thus be interpreted as inconclusive and needs further exploration.

By adjusting for important confounders which have not previously been included in studies of mental health and diet, this present study corroborates the finding that ‘unhealthy’ dietary patterns are associated with depression and anxiety [34,59,60]. One possible causal

pathway through which unhealthy foods such as processed (i.e., “foods that are altered to add or introduce substances that substantially change their nature or use”) and ultra-processed foods (i.e., “industrial formulations, usually made mainly or solely from industrial ingredients, which contain little or no whole food”) [61] may negatively impact mental health is that of inflammatory reactions and oxidative stress [62,63]. Interestingly, in this study, processed plant-based foods such as meat replacements did not load strongly on the junk food component but actually showed high component loadings in the plant food component. However, future research is needed to understand whether these foods reflect healthier dietary patterns, particularly given the rise of consumer demand for plant-based processed foods. For example, the development and application of a dietary screening measure that captures these foods in more detail may render a better understanding of these emerging dietary patterns.

The prevalence of clinically-relevant levels of depression and anxiety is high in this sample of 339 undergraduate students. These prevalence rates are in line with findings from previous research on the mental health of students indicating that the prevalence of mental health issues is higher among university students than in the general population [12,64]. There are several hypotheses why this may be the case. The typical age-of-onset of many psychiatric disorders overlaps with entry into university [11]; and the transition into university presents a stressful life event which is accompanied by homesickness, potentially social isolation, financial burden and pressure, and stress – all of which are risk factors for the development of depression and anxiety [65].

Conversely, more than half of the participants also report their overall QoL to be either very good or excellent. While this may at first seem counterintuitive, this is actually in line with the concept of QoL being a measure of a full continuum of (mental) wellbeing wherein the

presence of symptoms of a disorder such as depression and anxiety merely present one dimension. It has been found, for example, that factors such as self-esteem or social support mitigate the role of depressive symptoms on QoL [66]. Fahy et al. also found that the strongest predictors for QoL in people with severe mental illness were unmet basic, social, and functional needs (in combination with symptom severity)[67]. Thus, assessing QoL in addition to screening for depression and anxiety provides a more complete picture of mental wellbeing and its associated factors in this study.

The possibility of reverse causality is another important consideration that researchers have identified [30] with one prospective cohort study providing probable evidence for reverse causality between depression and a healthy diet pattern [68]. Because dietary changes are perceived as a means to shape health, it can be hypothesized that a change in dietary behaviour could follow the onset of mental health issues as a form of 'self-medication'. Conversely, the 'self-medication' may also take on the form of an unhealthy diet consistent of foods high in sugar and fat to feel instant gratification [69].

4.2. Strengths and limitations

This study utilizes a biopsychosocial conceptual understanding of the relationship between exposure and outcome and the inclusion of confounding variables that goes beyond a narrow biomedical approach. The present study builds on previous studies on this topic. Nevertheless, in order to eliminate temporal ambiguity, confounding, and response biases, more sophisticated study designs are needed in future investigations.

The relatively small sample size and the associated lack of power means that some effects of the explanatory variables may have remained uncovered in this study and that an

underestimation for these effects was likely present. This may have been amplified by the finding that self-reported data on diet typically leads to an underestimation of associations [58].

In this present study, all participants were undergraduate students. The external validity of this study beyond the student population is thus limited as university students differ from their non-student peers and the general population in several characteristics, e.g., in terms of socioeconomic backgrounds, lifestyle behaviours, or substance use [70,71]. In relation to the general population of undergraduates at the university, this sample was, however, fairly representative as its sociodemographic composition was comparable with that of the overall undergraduate student population. It is also important to note that across the continuum of depressive and anxiety symptoms, eating behaviours may differ (e.g., individuals with major depressive disorder often suffer from very reduced appetite and their overall food intake may be severely decreased). Thus, the findings of this study may not apply to individuals suffering from major depressive and anxiety disorders as this study was not conducted on a clinical sample.

All collected information was exclusively self-reported which introduces non-response, reporting, and recall biases. In this study, the primary interest was to assess diet patterns rather than exact nutrient intake. Self-reported diet data have been deemed adequate and superior to non-self-reported measures such as biomarkers especially when analyzing diet patterns as they provide more complete information on the composition of the overall diet [58]. Given that dietary screeners are less burdensome on participants than methods like repeated 24-hr recalls while still providing sufficient information on food intake, we chose to use the DSQ as measure for diet [48]. Its limitations include that it does not allow for conclusions about the actual amount of food intake nor does it capture the full range of foods in one's diet. To mitigate the subjectivity and biased information from self-reported mental health issues, this study included

validated screening instruments (one-item QoL scale, PHQ-9, GAD-7). While answers are still self-reported, these measures have been extensively validated.

It is also important to note that the different measures in this study assessed variables with different time frames. More specifically, the DSQ asked about food intake within the past 30 months whereas the PHQ-9 and GAD-7 assessed symptoms in the past two weeks. Other items evaluating covariables did not consider a specific time frame. Hence, based on the time-frames of the measures and the cross-sectional study design, inferences can only be made about the prevalence of the exposures and the outcomes and their degree of association at one point in time.

4.3. Conclusions and future directions

We show that plant foods are positively associated with mental health outcomes but this association is attenuated after adjusting for other variables in our biopsychosocial model. We further found no relationship between categories of certain diet preferences such as vegetarian or vegan and mental health. These findings support approaches in nutritional epidemiology that employ dietary pattern analyses. By taking a more sophisticated approach to covariate selection and dietary assessment, our findings add to the evidence that contrasts a widely accepted, albeit outdated, perception of vegetarians and vegans as unhealthy individuals at risk for nutrient deficiency [72,73]. This study provides a preliminary indication that the 'win-win' situation for planetary and somatic health of predominantly plant-based diets is not a 'win-win-lose' situation for mental health. Further research will be needed to confirm or refute this finding and would benefit from the inclusion of socioeconomic and cultural determinants as additional covariates of interest. For example, the issue of food security greatly impacts one's ability to access healthy

foods and has been associated with major depressive disorder in US women [74]. In addition, the ability to procure culturally-appropriate foods, which has been nearly eliminated by a colonial food system, is an issue of great extent for Indigenous communities and food traditions across the globe. How this may interact with mental health is of great importance and has been neglected in the public health literature at this point. Moreover, most of the studies on this topic have thus far have been conducted in North America, Europe, or Australia. Insights from countries and cultures other than Western nations would be helpful in understanding cross-cultural differences. Integrating research from social sciences, community action and participatory research, and findings from qualitative studies would also play a pivotal role in understanding the complex relationships under investigation.

Acknowledgements

We are grateful for the support of David Gill and the University of British Columbia's SEEDS program who guided this project in an encouraging way and substantially facilitated the integrated knowledge translation and community-based approach of this research. We would also like to extend our gratitude to the members of the stakeholder group, most notably Melissa Baker-Wilson and David Speight for their enthusiasm, support, and open-mindedness. It was a true pleasure to be surrounded by these like-minded, visionary change makers.

References

1. Willett W, Rockström J, Loken B, Springmann M, Lang T, Vermeulen S, et al. Food in the Anthropocene: the EAT–Lancet Commission on healthy diets from sustainable food systems. Lancet. 2019 Feb 2;393(10170):447–92.

2. Whiteford HA, Degenhardt L, Rehm J, Baxter AJ, Ferrari AJ, Erskine HE, et al. Global burden of disease attributable to mental and substance use disorders: findings from the Global Burden of Disease Study 2010. Lancet. 2013 Nov 9;382(9904):1575–86.

3. Demyttenaere K, Bruffaerts R, Posada-Villa J, Gasquet I, Kovess V, Lepine Jp, et al. Prevalence, severity, and unmet need for treatment of mental disorders in the World Health Organization World Mental Health Surveys. J Am Med Assoc. 2004;291(21):2581–90.

4. Somers JM, Goldner EM, Waraich P, Hsu L. Prevalence and incidence studies of anxiety disorders: a systematic review of the literature. Can J Psychiatry. 2006 Feb 1;51(2):100–13.

5. DeSilva MJ, McKenzie K, Harpham T, Huttly SRA. Social capital and mental illness: a systematic review. J Epidemiol Community Health. 2005 Aug 1;59(8):619–27.

6. Breslau J, Lane M, Sampson N, Kessler RC. Mental disorders and subsequent educational attainment in a US national sample. J Psychiatr Res. 2008 Jul 1;42(9):708–16.

7. Trautmann S, Rehm J, Wittchen H-U. The economic costs of mental disorders: Do our societies react appropriately to the burden of mental disorders? EMBO Rep. 2016 Sep 1;17(9):1245–9.

8. Alonso J, Angermeyer MC, Bernert S, Bruffaerts R, Brugha TS, Bryson H, et al. Disability and quality of life impact of mental disorders in Europe: results from the European Study of the Epidemiology of Mental Disorders (ESEMeD) project. Acta Psychiatr Scand. 2004;109(s420):38–46.

9. Burcusa SL, Iacono WG. Risk for recurrence in depression. Clin Psychol Rev. 2007;27(8):959–85.

10. Gibb SJ, Fergusson DM, Horwood LJ. Burden of psychiatric disorder in young adulthood and life outcomes at age 30. Br J Psychiatry. 2010;197(2):122–7.

11. Kessler RC, Amminger GP, Aguilar-Gaxiola S, Alonso J, Lee S, Üstün TB. Age of onset of mental disorders: a review of recent literature. Curr Opin Psychiatry. 2007 Jul;20(4):359.

12. Blanco C, Okuda M, Wright C, Hasin DS, Grant BF, Liu S-M, et al. Mental Health of College Students and Their Non–College-Attending Peers: results from the National Epidemiologic Study on Alcohol and Related Conditions. Arch Gen Psychiatry. 2008 Dec 1;65(12):1429–37.

13. Prince JP. University student counseling and mental health in the United States: trends and challenges. Ment Health Prev. 2015 May 1;3(1):5–10.

14. Evans TM, Bira L, Gastelum JB, Weiss LT, Vanderford NL. Evidence for a mental health crisis in graduate education. Nat Biotechnol. 2018;36(3):282.

15. McGorry PD, Mei C. Early intervention in youth mental health: progress and future directions. Evid Based Ment Health. 2018 Nov 1;21(4):182–4.

16. Patton GC, Viner R. Pubertal transitions in health. Lancet. 2007 Mar 31;369(9567):1130–9.

17. Logan AC, Jacka FN. Nutritional psychiatry research: an emerging discipline and its intersection with global urbanization, environmental challenges and the evolutionary mismatch. J Physiol Anthropol. 2014 Jul 24;33(1):22.

18. Sawyer SM, Afifi RA, Bearinger LH, Blakemore S-J, Dick B, Ezeh AC, et al. Adolescence: a foundation for future health. Lancet. 2012 Apr 28;379(9826):1630–40.

19. Flanagan R. More than 3 million Canadians vegetarian or vegan: study [Internet]. CTV News. 2018 [cited 2019 Mar 13]. Available from: https://www.ctvnews.ca/canada/more-than-3-million-canadians-vegetarian-or-vegan-study-1.4027606

20. Thomson A. “Mind-blowing”: Survey finds most vegans, vegetarians in Canada are under 35 [Internet]. CTV News. 2018 [cited 2019 Mar 13]. Available from: https://www.ctvnews.ca/health/mind-blowing-survey-finds-most-vegans-vegetarians-in-canada-are-under-35-1.3841041

21. The Nielsen Company. Plant-based proteins are gaining dollar share among North Americans [Internet]. Nielsen. 2017 [cited 2019 Mar 13]. Available from: http://www.nielsen.com/ca/en/insights/news/2017/plant-based-proteins-are-gaining-dollar-share-among-north-americans

22. Weersink A, Massow M von, Gallant M. Meat consumption is changing but it’s not because of vegans [Internet]. The Conversation. 2019 [cited 2019 Mar 13]. Available from: http://theconversation.com/meat-consumption-is-changing-but-its-not-because-of-vegans-112332

23. Derbyshire EJ. Flexitarian diets and health: a review of the evidence-based literature. Front Nutr. 2016;3:55.

24. Beezhold B, Johnston CS, Daigle DR. Vegetarian diets are associated with healthy mood states: a cross-sectional study in seventh day adventist adults. Nutr J. 2010;9(1):26.

25. Beezhold B, Radnitz C, Rinne A, DiMatteo J. Vegans report less stress and anxiety than omnivores. Nutr Neurosci. 2015 Oct 1;18(7):289–96.

26. Baines S, Powers J, Brown WJ. How does the health and well-being of young Australian vegetarian and semi-vegetarian women compare with non-vegetarians? Public Health Nutr. 2007 May;10(5):436–42.

27. Burkert NT, Muckenhuber J, Großschädl F, Rásky É, Freidl W. Nutrition and health - The association between eating behavior and various health parameters: a matched sample study. PLOS One. 2014 Feb 7;9(2):e88278.

28. Dobersek U, Wy G, Adkins J, Altmeyer S, Krout K, Lavie CJ, et al. Meat and mental health: a systematic review of meat abstention and depression, anxiety, and related phenomena. Crit Rev Food Sci Nutr. 2020 Apr 20;0(0):1–14.

29. Hibbeln JR, Northstone K, Evans J, Golding J. Vegetarian diets and depressive symptoms among men. J Affect Disord. 2018;225:13–7.

30. Michalak J, Zhang XC, Jacobi F. Vegetarian diet and mental disorders: Results from a representative community survey. Int J Behav Nutr Phys Act. 2012 Jun 7;9:67.

31. Hu FB. Dietary pattern analysis: a new direction in nutritional epidemiology. Curr Opin Lipidol. 2002;13(1):3–9.

32. Satija A, Bhupathiraju SN, Rimm EB, Spiegelman D, Chiuve SE, Borgi L, et al. Plant-based dietary patterns and incidence of type 2 Diabetes in US men and women: Results from three prospective cohort studies. PLOS Med. 2016 Jun 14;13(6):e1002039.

33. Lai JS, Hiles S, Bisquera A, Hure AJ, McEvoy M, Attia J. A systematic review and meta-analysis of dietary patterns and depression in community-dwelling adults. Am J Clin Nutr. 2014 Jan 1;99(1):181–97.

34. Lassale C, Batty GD, Baghdadli A, Jacka F, Sánchez-Villegas A, Kivimäki M, et al. Healthy dietary indices and risk of depressive outcomes: a systematic review and meta-analysis of observational studies. Mol Psychiatry. 2018 Sep 26;1.

35. Li Y, Lv M-R, Wei Y-J, Sun L, Zhang J-X, Zhang H-G, et al. Dietary patterns and depression risk: a meta-analysis. Psychiatry Res. 2017 Jul 1;253:373–82.

36. Diener E, Seligman MEP. Very happy people. Psychol Sci. 2002 Jan 1;13(1):81–4.

37. Karatsoreos IN, McEwen BS. Psychobiological allostasis: resistance, resilience and vulnerability. Trends Cogn Sci. 2011 Dec 1;15(12):576–84.

38. Paluska SA, Schwenk TL. Physical activity and mental health. Sports Med. 2000 Mar 1;29(3):167–80.

39. Tiggemann M. Body dissatisfaction and adolescent self-esteem: prospective findings. Body Image. 2005 Jun 1;2(2):129–35.

40. Van Kim NA, Nelson TF. Vigorous physical activity, mental health, perceived stress, and socializing among college students. Am J Health Promot. 2013 Sep 1;28(1):7–15.

41. Martin W, Dixon BJ, Thomas H. Enhancing mental well-being. In: The Handbook of Stress and Health [Internet]. 1st ed. John Wiley & Sons, Ltd; 2017 [cited 2019 Apr 9]. p. 459–71. Available from: http://www.onlinelibrary.wiley.com/doi/abs/10.1002/9781118993811.ch28

42. Aarø LE, Laberg JC, Wold B. Health behaviours among adolescents: towards a hypothesis of two dimensions. Health Educ Res. 1995 Mar 1;10(1):83–93.

43. Conklin AI, Forouhi NG, Surtees P, Khaw K-T, Wareham NJ, Monsivais P. Social relationships and healthful dietary behaviour: evidence from over-50s in the EPIC cohort, UK. Soc Sci Med. 2014 Jan 1;100:167–75.

44. Leigh Gibson E. Emotional influences on food choice: sensory, physiological and psychological pathways. Physiol Behav. 2006 Aug 30;89(1):53–61.

45. Neely E, Walton M, Stephens C. Young people’s food practices and social relationships. A thematic synthesis. Appetite. 2014 Nov 1;82:50–60.

46. Neumark-Sztainer D, Paxton SJ, Hannan PJ, Haines J, Story M. Does body satisfaction matter? Five-year longitudinal associations between body satisfaction and health behaviors in adolescent females and males. J Adolesc Health. 2006 Aug 1;39(2):244–51.

47. Weidner G, Kohlmann C-W, Dotzauer E, Burns LR. The effects of academic stress on health behaviors in young adults. Anx Stress Coping. 1996;9(2):123–33.

48. Thompson FE, Midthune D, Kahle L, Dodd KW. Development and Evaluation of the National Cancer Institute’s Dietary Screener Questionnaire Scoring Algorithms123. J Nutr. 2017 Jun;147(6):1226–33.

49. Cella D, Riley W, Stone A, Rothrock N, Reeve B, Yount S, et al. The Patient-Reported Outcomes Measurement Information System (PROMIS) developed and tested its first wave of adult self-reported health outcome item banks: 2005–2008. J Clin Epidemiol. 2010 Nov 1;63(11):1179–94.

50. Arroll B, Goodyear-Smith F, Crengle S, Gunn J, Kerse N, Fishman T, et al. Validation of PHQ-2 and PHQ-9 to screen for major depression in the primary care population. Ann Fam Med. 2010 Jul 1;8(4):348–53.

51. Beard C, Hsu KJ, Rifkin LS, Busch AB, Björgvinsson T. Validation of the PHQ-9 in a psychiatric sample. J Affect Disord. 2016 Mar 15;193:267–73.

52. Keum BT, Miller MJ, Inkelas KK. Testing the factor structure and measurement invariance of the PHQ-9 across racially diverse U.S. college students. Psychol Assess. 2018;30(8):1096–106.

53. Kroenke K, Spitzer RL, Williams JBW. The PHQ-9. J Gen Intern Med. 2001 Sep;16(9):606–13.

54. Kroenke K, Spitzer RL, Williams JBW, Monahan PO, Löwe B. Anxiety disorders in primary care: prevalence, impairment, comorbidity, and detection. Ann Intern Med. 2007 Mar 6;146(5):317.

55. Spitzer RL, Kroenke K, Williams JBW, Löwe B. A brief measure for assessing generalized anxiety disorder: the GAD-7. Arch Intern Med. 2006 May 22;166(10):1092–7.

56. Enders CK. Applied Missing Data Analysis. Methodology in the Social Sciences Series. Guilford Press; 2010.

57. Bailey RL, Mitchell DC, Miller CK, Still CD, Jensen GL, Tucker KL, et al. A dietary screening questionnaire identifies dietary patterns in older adults. J Nutr. 2007 Feb 1;137(2):421–6.

58. Subar AF, Freedman LS, Tooze JA, Kirkpatrick SI, Boushey C, Neuhouser ML, et al. Addressing current criticism regarding the value of self-report dietary data. J Nutr. 2015 Dec 1;145(12):2639–45.

59. Jacka FN, Pasco JA, Mykletun A, Williams LJ, Hodge AM, O'Reilly SL, et al. Association of Western and traditional diets with depression and anxiety in women. Am J Psychiatry. 2010 Mar 1;167(3):305–11.

60. Jacka FN, Mykletun A, Berk M, Bjelland I, Tell GS. The association between habitual diet quality and the common mental disorders in community-dwelling adults: the Hordaland Health Study. Psychosom Med. 2011 Jul;73(6):483–90.

61. Moubarac J-C, Batal M, Martins APB, Claro R, Levy RB, Cannon G, et al. Processed and ultra-processed food products: consumption trends in Canada from 1938 to 2011. Can J Diet Pract Res. 2014 Mar 1;75(1):15–21.

62. Kaplan BJ, Rucklidge JJ, Romijn A, McLeod K. The emerging field of nutritional mental health: inflammation, the microbiome, oxidative stress, and mitochondrial function. Clin Psychol Sci. 2015 Nov 1;3(6):964–80.

63. Nettleton JA, Steffen LM, Mayer-Davis EJ, Jenny NS, Jiang R, Herrington DM, et al. Dietary patterns are associated with biochemical markers of inflammation and endothelial activation in the Multi-Ethnic Study of Atherosclerosis (MESA). Am J Clin Nutr. 2006 Jun 1;83(6):1369–79.

64. Statistics Canada. Mental Health and Well-being profile, Canadian Community Health Survey (CCHS), by age group and sex, Canada and provinces [Internet]. 2018 [cited 2019 Apr 16]. Available from: https://www150.statcan.gc.ca/t1/tbl1/en/tv.action?pid=1310009201

65. Beiter R, Nash R, McCrady M, Rhoades D, Linscomb M, Clarahan M, et al. The prevalence and correlates of depression, anxiety, and stress in a sample of college students. J Affect Disord. 2015 Mar 1;173:90–6.

66. Kuehner C, Buerger C. Determinants of subjective quality of life in depressed patients: the role of self-esteem, response styles, and social support. J Affect Disord. 2005 Jun 1;86(2):205–13.

67. Fahy T, Kent A, Tattan T, Horn EV, White I. Predictors of quality of life in people with severe mental illness: study methodology with baseline analysis in the UK 700 trial. Br J Psychiatry. 1999 Nov;175(5):426–32.

68. Le Port A, Gueguen A, Kesse-Guyot E, Melchior M, Lemogne C, Nabi H, et al. Association between dietary patterns and depressive symptoms over time: a 10-year follow-up study of the GAZEL cohort. PLOS One. 2012;7(12):e51593.

69. Jantaratnotai N, Mosikanon K, Lee Y, McIntyre RS. The interface of depression and obesity. Obes Res Clin Pract. 2017 Jan 1;11(1):1–10.

70. Bailey MJ, Dynarski SM. Gains and Gaps: Changing Inequality in U.S. College Entry and Completion [Internet]. National Bureau of Economic Research; 2011 Dec [cited 2019 Apr 14]. Report No.: 17633. Available from: http://www.nber.org/papers/w17633

71. O’Malley PM, Johnston LD. Epidemiology of alcohol and other drug use among American college students. J Stud Alcohol Suppl. 2002 Mar 1;(s14):23–39.

72. Leitzmann C. Vegetarian nutrition: past, present, future. Am J Clin Nutr. 2014;100(suppl_1):496S-502S.

73. Sabaté J. The contribution of vegetarian diets to health and disease: a paradigm shift? Am J Clin Nutr. 2003 Sep 1;78(3):502S-507S.

74. Beydoun MA, Wang Y. Pathways linking socioeconomic status to obesity through depression and lifestyle factors among young US adults. J Affect Disord. 2010 Jun 1;123(1):52–63.

Supporting information

Questionnaire

Questionnaire

Click here to access/download
Supporting Information
Supporting information_Questionnaire.pdf
